# Molecular Characterization Reveals Recombination and Host Exchange of Adenoviruses in Migratory Birds in China

**DOI:** 10.1155/tbed/3030377

**Published:** 2025-09-27

**Authors:** Jie Li, Xiaofang Ma, Ru Jia, Shanrui Wu, Yisong Li, Lan Wang, Yeshun Fan, Ying Wang, Dianfeng Chu, Yihua Wang, Guogang Zhang, Jie Liu

**Affiliations:** ^1^School of Public Health, Qingdao University, Qingdao, Shandong, China; ^2^Qingdao Municipal Center for Disease Control and Prevention, Institute of Disinfection and Vector Borne Disease Control, Qingdao, Shandong, China; ^3^Ecology and Nature Conservation Institute, Chinese Academy of Forestry, Beijing, China; ^4^Yebio BioEngineering Co. Ltd. of Qingdao, Qingdao, Shandong, China

**Keywords:** adenovirus, bar-headed goose adenovirus, host exchange, migratory birds, PCR, recombination

## Abstract

Migratory birds, because of their migration and roosting characteristics, can serve as major vectors for long-distance transmission, recombination, and evolution of adenoviruses. China is one of the countries possessing the widest variety of birds in the world, with the global migration routes covering almost the entire territory. However, avian adenoviruses haven't been systematically studied. In the current study, PCR-based molecular methods were used to characterize the adenoviruses in 38 migratory bird species from nine provinces in China from October 2020 to March 2021. Aviadenoviruses (11.4%, 79/690) were predominantly detected, followed by siadenoviruses (6.2%, 43/690) and barthadenoviruses (1.3%, 9/690). Phylogenetic analysis demonstrated about half of the aviadenoviruses clustered with Duck adenovirus 2, revealing potential association with poultry animals. A high portion (67.2%, 88/131) of the DNA polymerase sequences had <85% identity to any known sequences, indicating the potential presence of novel species, particularly in bar-headed goose where adenoviruses of all three genera were detected for the first time. The clustering of adenovirus sequences from different birds and regions in the same branch of the phylogenetic analysis suggested their close genetic relationships, indicating the transmission of adenoviruses across bird species. Host exchange and recombination events were observed, which might reflect the plasticity of these viruses and the mechanism for the emergence of novel viruses. The prevalence and characteristics of the adenoviruses in migratory birds demonstrated the wide distribution of novel adenovirus species and possible transmission between wild birds and domestic animals.

## 1. Introduction

Adenoviruses are medium-sized, nonenveloped, linear, and double-stranded DNA viruses in the *Adenoviridae* family. The genome size is about 27–48 kb and both ends have inverted terminal repeats (ITR) [1, 2]. Currently, adenoviruses are divided into six genera [3–5], including *Aviadenovirus*, *Barthadenovirus*, *Ichtadenovirus*, *Mastadenovirus*, *Siadenovirus*, and *Testadenovirus*.

Adenoviruses are widespread in many hosts, including humans, where human adenoviruses were first reported in the 1950s [6, 7]. Adenoviruses usually cause mild infections in humans, including upper or lower respiratory tract infections, gastrointestinal diseases, conjunctivitis, and other infrequent conditions such as hemorrhagic cystitis, hepatitis, et cetera. Adenovirus infections are more common in young children because their immune systems are still under development [8–10]. Other mammals, reptiles, and birds are frequent hosts of adenoviruses [11–14]. The high species diversity, migration, and unique adaptive immune system of birds enable a natural reservoir for adenovirus transmission and a genetic source for the evolution and emergence of novel adenoviruses [15]. A new species of adenovirus within a genus is defined as >10%–15% phylogenetic distance based on DNA-dependent DNA polymerase sequences [16]. A novel adenovirus discovered in mealy parrot (*Amazona farinosa*) in 2012 increased the severity of *Chlamydia psittaci* infection in the birds that was epidemiologically linked to an outbreak in humans through a pathogen augmentation mechanism [17, 18]. It indicated that cross-species transmission of zoonotic diseases might be associated with underlying viral infection in the relevant animals.

China is on three of the nine global migration routes of birds and possesses one-fifth of the migratory birds in the world. The East Asia-Australia migration route is the most important migration route for migratory birds in China, with the largest number and most variety. The global distribution of birds, annual long-distance migration, and habitual roosting in environments shared with other animals further enhance the cross-species transmission of viruses [12]. To our knowledge, the research on adenoviruses in China has mainly focused on adenovirus diseases in domesticated poultry; little is known about adenoviruses in wild birds. Shan et al. [19] sequenced the virome in the cloaca of both wild and breeding birds, and identified seven adenovirus genomes in seven different species of birds. No adenovirus was found in Anseriformes, which was the main migratory bird type interrogated in the study. A broad surveillance of adenovirus in migratory birds would help to understand the distribution of viruses, and inform the transmission risks and their pathogenic potential.

In the current study, we collected fecal samples at the major migratory bird habitats in China to investigate the prevalence and diversity of adenoviruses, then further performed phylogenetic analysis and analyzed the recombination events in adenoviruses.

## 2. Materials and Methods

### 2.1. Specimens

Fresh fecal samples were collected from Xingkai Lake in Heilongjiang, Cangzhou in Hebei, Longbao Nature Reserve in Qinghai, Grand View Pavilion and Dianchi Lake in Yunnan, Minjiang estuary in Fujian, Lingwu City in Ningxia, Wanghu Lake in Hubei, Tumuji National Nature Reserve in Inner Mongolia, and counties along the Yarlung Zangbo River in Xizang from October 2020 to March 2021. The locations where migratory birds gather and forage were selected for sample collection upon remote observation through a telescope. The researchers then slowly entered the areas and collected uncontaminated fresh fecal samples from the ground with cotton swabs. The samples were stored in a preservative solution (sodium chloride 0.9%, penicillin 0.2 mg/mL, streptomycin sulfate 2 mg/mL, and glycerol 20%) at 80°C until testing.

### 2.2. Nucleic Acid Extraction

Total nucleic acid was extracted with a Tianlong Viral DNA/RNA Extraction kit on anautomated Tianlong nucleic acid extraction system (Tianlong Technology Co., Ltd., Xi'an, China) or manually with QIAamp Fast DNA Stool Mini Kit (Qiagen, Hilden, Germany). Both kits have been widely evaluated and utilized. For manual extraction, the samples were pretreated with a bead-beating step that was previously established to improve the yield of nucleic acid extraction [20, 21]. Briefly, 200 µL of fecal suspension was mixed with 1 mL of InhibitEX Buffer, and beaten with glass beads of 212–300 µm (Sigma–Aldrich, St. Louis, USA) in the OMNI Bead Ruptor 24 Elite (Kennesaw GA, USA) for four times at 8 m/s for 30 sec with an interval of 20 sec.The mixture was then heat treated at 95°C for 5 min. The InhibitEX buffer was spiked with external controls, MS2 bacteriophage and Phocine Herpesvirus at 10^7^ and 10^6^ copies per sample, respectively, to monitor the extraction and amplification efficiency. After centrifugation at 12,000 rpm, 600 µL of supernatant was recovered and extracted following the manufacturer's instructions. Nucleic acid was eluted with 200 µL buffer ATE and stored at 80°C until PCR.

### 2.3. PCR Primer Design and PCR Amplification

The adenovirus DNA polymerase gene was amplified by nested PCR using published Pan-adenovirus primers (Table 1) to amplify a sequence of about 300 bp with a Touchdown setup [22]. For the first round of PCR amplification, the 20 µL reaction contained 10 µL of Taq Plus Master Mix II (Vazyme, Nanjing, China), 20 pmol of each first-round primer, 1 µL of nucleic acid extract, and nuclease-free water up to 20 µL. The Touchdown PCR was programed as follows: initial denaturation at 95°C for 3 min, 14 cycles of 95°C for 15 s and 60–46°C for 60 s, followed by 30 cycles of 95°C for 15 s, 46°C for 30 s, and 72°C for 60 s, and a final extension at 72°C for 10 min. For the second-round of amplification, the second round primers were used at the same concentration, and 2 µL of the PCR reaction from the first round was added. The reaction conditions were the same as in the first round except that the cycle number was increased to 40 for the step of 95°C for 15 s, 46°C for 30 s, and 72°C for 60 s.

Based on the aviadenovirus sequences available from the NCBI library, one primer targeting the hexon gene was designed with Primer3 v0.4.0 and combined with a set of published primers to formulate a nested PCR (Table 1) to amplify approximately 1000 bp of the *Aviadenovirus* genus. The reaction mixture was the same as that for DNA polymerase and the cycling conditions for both the first and second rounds of amplification were set as follows: initial denaturation at 95°C for 3 min, followed by 30 cycles of 95°C for 15 s, 53°C for 30 s, and 72°C for 60 s, and a final extension at 72°C for 10 min. Likewise, a pair of primers (Table 1) targeting the hexon gene was designed with Primer3 v0.4.0 to amplify approximately 1000 bp of the *Siadenovirus* genus. The reaction mixture was the same as that for DNA polymerase and the cycling conditions were set as follows: initial denaturation at 95°C for 3 min, followed by 45 cycles of 95°C for 15 s, 56°C for 30 s, and 72°C for 60 s, and a final extension at 72°C for 10 min.

The birds' mitochondrial cytochrome oxidase subunit I (COX-I or COI) gene was amplified using a set of published primers [26], combined with an additional modified reverse primer (Table 1) to amplify a sequence of about 450 bp. The 20 µL reaction contained 10 µL of Taq Plus Master Mix II, 4 pmol of each primer, 1 µL of nucleic acid extract, and nuclease-free water up to 20 µL. The PCR cycling conditions were set as follows: initial denaturation at 95°C for 3 min, followed by 45 cycles of 95°C for 15 s, 58°C for 30 s, and 72°C for 60 s, and a final extension at 72°C for 10 min.

Positive (synthetic DNA fragment of known sequences targeted by PCR, Tsingke, Qingdao, China) and negative controls were included for each PCR setup to assess the validity of the results. PCR products were run on 1% agarose gel, and the products with the expected amplicon length were sequenced by Sangon (Qingdao, China). When poor sequencing quality implied possible mixed adenoviruses, next-generation sequencing with Illumina MiSeq was performed (Sangon, Qingdao, China). The sequences were compared with known sequences by BLAST and uploaded to the NCBI database (accession numbers from PP319046 to PP319176 for DNA polymerase gene sequences; accession numbers from PP331810 to PP331835 for hexon gene sequences).

### 2.4. Phylogenetic Analysis and Gene Characterization

The maximum likelihood tree was generated by MAFFT software [27] for sequence alignment and PhyML software [28] for the acquired amino acid sequences of the adenovirus DNA polymerase gene to determine the phylogeny of the adenoviruses. NeighborNet network was built with SplitsTree v4.18.3 under 1000 replicates using nucleic acid sequences of DNA polymerase gene from representative adenoviruses. Tanglegram was generated using Dendroscope v3.8.2 with a subset of paired DNA polymerase gene sequences and hexon gene sequences [29].

### 2.5. Recombination Analysis

Recombination analysis was performed based on the adenovirus hexon gene, using the RDP, Bootscan, MaxChi, GENECONV, Siscan, Chimaera, and 3Seq methods included in the RDP4 program [30]. As shown in Figure S1, the schematic sequence display contained breakpoint information, recombination regions, and background sequences, and the plot display showed the potential recombination regions and the homology of the parent with the recombination sequence. Based on the criteria established by Athukorala et al., [31] an event with significant *p*-values from at least two of the above methods was considered a possible recombination event.

### 2.6. Cluster Analysis

Clustering analysis was based on DNA polymerase gene, using CD-HIT-EST function module in cd-hit software [32], with sequence consistency threshold set at 0.85 and coverage rate at 0.9 for DNA clustering.

### 2.7. Statistical Analysis

The prevalence of adenoviruses was chosen for the descriptive analysis, and the differences in adenovirus detection across regions were compared with a chi-square test using SPSS v26.0 (IBM). Two-tailed *p*-values were calculated, and values of <0.05 were considered statistically significant.

## 3. Results

### 3.1. Adenovirus Detection and Bird Identification

**Figure 1:**
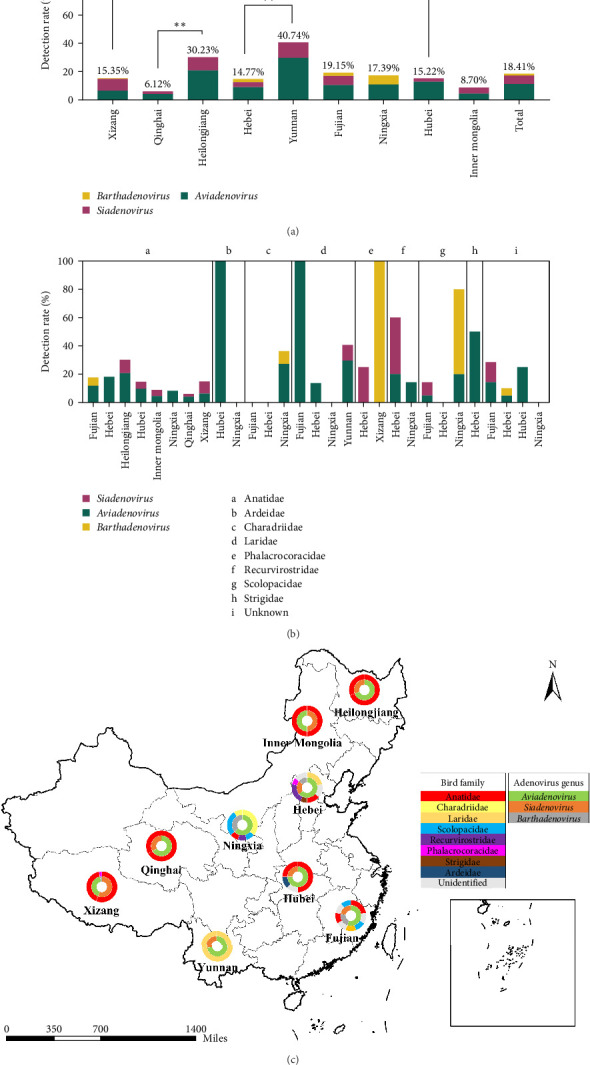
The distribution of adenoviruses in a variety of migratory birds at nine habitat sites in China. (a) Adenovirus detection across migratory bird habitat sites. (*⁣*^*∗∗*^ indicate statistically significant difference). (b) Adenovirus detection in different bird families. (c) Geographical distribution of adenoviruses and their hosts. The inner circle represents the adenovirus genera, and the outer circle represents the bird families.

A total of 690 samples were collected from 38 species of migratory birds of 11 families and six orders at nine habitats in China (Table S1), including Anseriformes, Charadriiformes, Suliformes, Pelecaniformes, Gruiformes, and Strigiformes, with 58 unidentifiable by COI DNA barcode. Overall 127 (18.4%) were detected positive for adenoviruses in eight families of birds, including predominantly aviadenoviruses (78, 11.3%), siadenoviruses (44, 6.2%), and barthadenoviruses (9, 1.3%). The overall adenovirus detection varied from site to site (Figure 1a), with the highest in Yunnan (40.7%), followed by Heilongjiang (30.2%), and the lowest in Qinghai (6.1%, *p*  < 0.05). Aviadenoviruses were detected at all nine sites, ranging from 29.6% in Yunnan to 4.0% in Qinghai. Siadenoviruses were detected at eight sites, with a similar prevalence in Yunnan, Xizang, and Heilongjiang. Barthadenoviruses were detected at only four sites with a relatively lower prevalence compared to the other two genera. No siadenovirus was found in Ningxia while it had the highest barthadenoviruses detected (6.5%). Among the 34 adenovirus positive samples collected from 10 counties and cities along the Yarlung Tsangpo River in Xizang, no statistically different distribution was observed (*p*=0.310). Next generation sequencing results revealed that mixed adenoviruses were detected in three samples, including barthadenovirus and aviadenovirus in one sample from Ningxia, two different species of aviadenoviruses and barthadenovirus in another sample from Ningxia, siadenovirus and aviadenovirus in one sample from Hubei.

As shown in Figure 1 and Table S1, the bird populations interrogated in this study were diverse in Fujian, Hebei, and Ningxia, while the remaining sites had mostly a single species with rare exceptions, such as exclusively Anatidae in Heilongjiang and Inner Mongolia. The detection rate of adenoviruses varied by bird family, that is, 33.3%, 32.9%, 26.7%, 21.9%, and 16.1% for Recurvirostridae, Laridae, Charadriidae, Scolopacidae, and Anatidae, respectively. Anatidae distributed the most broadly at eight of the nine sites with variable detection of all three adenovirus genera. Other families of birds showed limited locations and more distinct detection rates of adenoviruses among the relevant sites. The host was unidentifiable in seven adenovirus positive samples. Excluding the bird species with sample size <10, Laridae in Yunnan had the highest detection of overall adenoviruses (40.7%), aviadenoviruses (29.6%), and siadenoviruses (11.1%), while Charadriiformes in Ningxia had the highest barthadenoviruses (16.0%).

### 3.2. Phylogenetic Analysis and Genetic Characterization

**Figure 2:**
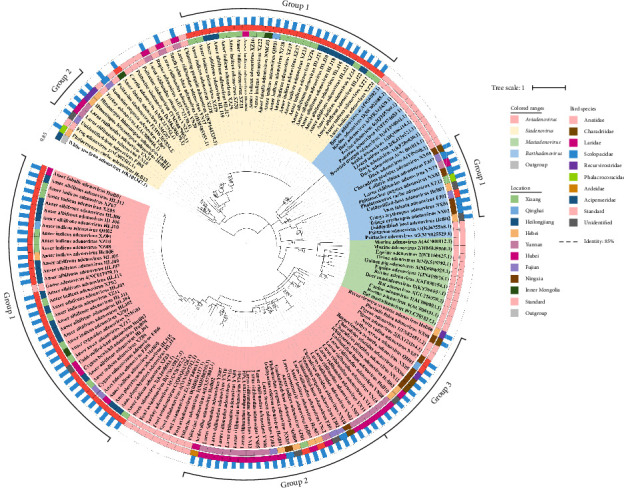
The phylogenetic tree shows the evolutionary relationships of the detected avian adenoviruses with other adenoviruses. A maximum likelihood (ML) tree was constructed using the amino acid sequence of the partial DNA polymerase gene. The color of the shade indicates the different genera, that is, green for *Mastadenovirus*, blue for *Barthadenovirus*, red for *Aviadenovirus*, and yellow for *Siadenovirus*. Similarly, bird species (middle circle) and locations (inner) are included in the legend. The column in blue (outer) indicates the sequence identity of the adenoviruses in this study with the adenovirus sequences in the NCBI database while the dotted line shows the identity of 85%. Sequence information of adenoviruses is listed in Table S2.

The maximum likelihood tree was constructed with amino acid sequences of partial DNA polymerase (Figure 2). The phylogenetic tree clearly showed that most of the aviadenoviruses detected in this study were divided into three groups. One group consisting of sequences exclusively from Anatidae at different locations except one host unidentified in Hubei, clustered with Goose adenovirus 4 and the Duck adenovirus 2. The other two groups formed distinct new branches with Laridae predominant for one group and a more diverse bird type distribution for the other. The criterion for identification of novel species within an adenovirus genus requires sequence identity to the closest related adenoviral species be lower than 85%–90% [33]. Worth noting was that the majority of the sequences in the first cluster (32/35) showed >85% identity with known adenovirus sequences, with 30 identified as *Aviadenovirus anseris* (compared to Goose adenovirus 4) and two as *Aviadenovirus anatis* (compared to Duck adenovirus 2). On the opposite, the BLAST identity of the sequences in the two new clusters were all <85% (67.4%−84.5%) except three sequences, with the best hit against fowl adenovirus (FAdV). Interestingly, all aviadenovirus sequences (*n* = 16) from Yunnan and 6/7 from both Ningxia and Hebei belonged to one or both new clusters. Due to the low identity to the known sequences, their genotypes could not be reliably identified.

The siadenoviruses formed two main clusters. One exclusively contained the sequences of Anatidae (26) from different locations, which were clustered with frog adenovirus at a phylogenetic distance. Six sequences from birds of three families in Yunnan, Hebei, and Fujian formed a new cluster. The most striking observation was that all the siadenovirus sequences had <85% identity to any known sequences. Most (8/9) of the barthadenoviruses were clustered together on a new branch, showing <85% identity to the known sequences except one, and one from Ningxia was placed on the most ancient branch of the genus.

The NeighborNet analysis was further performed with the nucleotide sequences of DNA polymerase from known bird hosts, together with the relevant sequences downloaded from NCBI to explore the host specificity and potential host exchange or recombination. The adenoviruses were classified at the molecular level into four major genera including *Barthadenovirus*, *Aviadenovirus*, *Mastadenovirus*, and *Siadenovirus*, and then further into several lineages (Figure S2). The evolutionary relationships of adenoviruses became evident through phylogenetic trees. The detected adenovirus sequences in this study had a high identity to each other in the *Siadenovirus* genus and were mainly grouped in the same sub-branch hosted by *Anser indicus* and *Anser albifrons*, which appeared to be a unique avian lineage of the *Siadenovirus* genus separating from the known lineages 1–4. Interestingly, one *Phalacrocorax carbo* adenovirus belonged to lineage 2, whereas the rest of the siadenoviruses in this study were independent of the known lineages. Most of the aviadenoviruses from Anseriformes studied were in the same lineage as Goose adenovirus 4 (lineage 2), including mostly *A. indicus* adenoviruses, two *Anas platyrhynchos* adenoviruses, and one *Aythya nyroca* adenovirus. Pigeon and FAdVs were categorized as lineage 1 and 3, respectively, and lineage 4 mainly consisted of Psittaciformes adenoviruses. Significantly, Charadriiformes adenoviruses were found to be categorized into two branches outside of the four known lineages. One branch included four families, including Laridae, Scolopacidae, Charadriidae, and Recurvirostridae, and was aggregated with three Anseriformes adenoviruses and one case of *Asio flammeus* adenovirus of Strigiformes. The other branch included only one *Recurvirostra avosetta* (Recurvirostridae) adenovirus and one *Pluvialis fulva* (Charadriidae) adenovirus of the Charadriiformes. The adenoviruses in the barthadenovirus group were found in more diverse hosts, with each cluster corresponding to certain species, such as bovine and deer adenoviruses in the same branch belonging to lineage 2. Lineage 3 divided into 3A and 3B. An adenovirus found in *Charadrius placidus* (Charadriiformes, Charadriidae) was categorized in lineage 3A. One *P. carbo* adenovirus and one *Calidris alpina* adenovirus in this study were positioned in lineage 2, which otherwise contained exclusively adenoviruses from reptiles. The remaining clustered together outside of the known lineages. More importantly, the NeighborNet analysis showed a reticulate structure, indicating that recombination was frequent across species, including many identified in the current study.

### 3.3. Recombination Analysis

**Figure 3:**
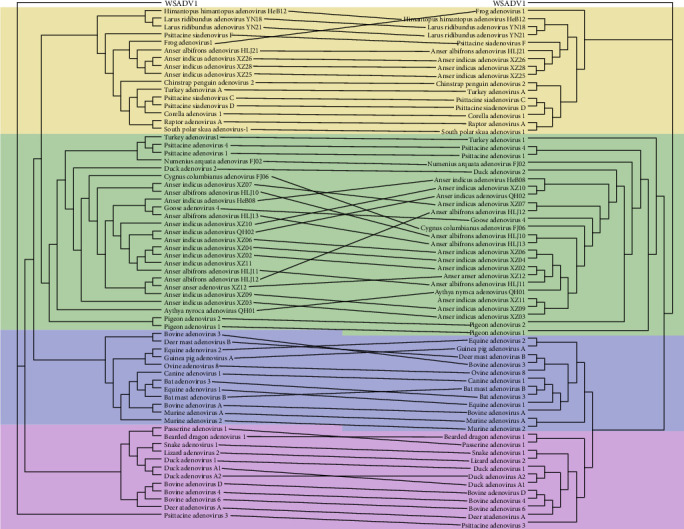
Coevolutionary tree of DNA polymerase and hexon genes. The nucleotide sequences of DNA polymerase and hexon genes were used to produce a phylogenetic tree of the adenovirus, and a tanglegram was formed using Dendroscope. The lines between the hexon (right) and DNA polymerase (left) represent their relationship. The color of the shade indicates the different genera, that is, blue for *Mastadenovirus*, purple for *Barthadenovirus*, green for *Aviadenovirus*, and yellow for *Siadenovirus*. White sturgeon adenovirus 1 (WSADV 1) was used as the outgroup.

The coevolutionary tree of DNA polymerase and hexon genes was built to compare their phylogenetic relationship to further characterize the recombination events. The hexon gene was characterized in a subset of 26 samples from a variety of bird species, including 13 from *A. indicus* (12) and *Anser anser* (1) in Xizang, five from *A. albifrons* in Heilongjiang, two from *A. indicus* and *A. nyroca* in Qinghai, two from *A. indicus* and *Himantopus himantopus* in Hebei, two from *Larus ridibundus* in Yunnan, and two from *Numenius arquata* and *Cygnus columbianus* in Fujian. Therefore, the coevolutionary tree of DNA polymerase and hexon genes was built based on these 26 samples (Figure 3), and additionally, recombination analysis was performed (Table 2 and Figure S1). For the majority of the sequences, the two genes corresponded to each other (Figure 3). However, cross-relation was observed intra-genus, which was particularly evident in the *Aviadenovirus* genus, where recombination events were also found in these sequences (Table 2). For example, *C. columbianus* adenovirus FJ06 was found to be a recombination of Duck adenovirus 2, frequently found in poultry, with *A. indicus* adenovirus XZ02 as the parental source. *Anser albifrons* adenovirus HLJ13 and *L. ridibundus* adenovirus YN21 were recognized as the recombination products with five out of seven different methods. Evidently, in the co-evolutionary trees, *C. columbianus* adenovirus FJ06 and *A. albifrons* adenovirus HLJ13 were among those crossed over.

### 3.4. Cluster Analysis of Potentially Novel Adenoviruses

Of the 131 sequences obtained with high quality, 88 sequences showed less than 85% identity to known adenovirus species. Cluster analysis was further performed to explore their phylogenetic relationship. The results of cluster analysis showed that 88 sequences possibly belonged to 39 distinct species (Table 3), including 22 species of *Aviadenovirus* (38 sequences), 12 species of *Siadenovirus* (41 sequences), and five species of *Barthadenovirus* (nine sequences). If requiring at least two sequences be available for each species, 10 species were identified, that is seven species of *Aviadenovirus*, two of *Siadenovirus*, and one of *Barthadenovirus*. The most common siadenovirus species had 29 sequences, exclusively from Anatidae at multiple locations including Xizang, Heilongjiang, Qinghai, Hubei, and Inner Mongolia. The one barthadenovirus species was found in Hebei, Ningxia, and Fujian. On the contrary, the nine sequences of the most predominant aviadenovirus species were all collected from Yunnan.

## 4. Discussion

By the most recent update, 109 adenovirus species with full genome sequenced have been accepted by ICTV while there have been a growing number of unspeciated and partially sequenced DNA polymerase or hexon genes. In the current study, close to one-fifth of the 690 fecal samples from a variety of migratory birds in nine provinces in China were found to be positive for adenovirus, with 58.3% being aviadenovirus, 33.9% siadenovirus, 5.5% barthadenovirus, and 2.4% mixed species. Most (61.4%, 78/127) of these adenoviruses originated from Anseriformes, with the rest mostly from Charadriiformes. This is different from the previous studies which found a higher detection rate of adenoviruses in Charadriiformes than in Anseriformes [34–36]. The phylogenetic analysis demonstrated the diversity within all three genera, including clusters completely separated from known sequences. The previous study in Australia showed a rich diversity of adenoviruses [37], with 42% novel sequences, including predominantly barthadenoviruses (40%), followed by siadenoviruses (34%) and aviadenoviruses (20%). While of the 14 new strains identified in Hungary, only one was classified as an established adenovirus species. All of the other strains would represent new adenovirus species with sequence agreement below the speciation criteria: 85%–90% [33]. A high portion (67.2%, 88/131) of the DNA polymerase sequences obtained in this study, including 95.4% (41/43) of siadenoviruses, 100% (*n* = 9) of barthadenoviruses, and 48.1% (38/79) of aviadenoviruses, had less than 85% identity with those from the NCBI database, indicating the potential presence of novel adenovirus species in China. Moreover, different from the recent findings of novel barthadenoviruses in Australia and Europe [33, 37], novel siadenovirus and aviadenovirus DNA polymerase sequences appeared to be much more abundant in our study.

Worth noting is that adenoviruses from three genera were detected in samples collected from *Anser indicus*. To our knowledge, there has not been any report on adenovirus detection in this bird species. Therefore, for the adenoviruses identified in *A. indicus*, we classify the aviadenoviruses clustered with Goose adenovirus 4 as Group 1 of *Aviadenovirus* in Figure 2 (e.g., *A. indicus* adenovirus XZ02, NCBI accession number PP319052.1 for DNA polymerase gene, PP331812.1 for hexon gene) as bar-headed Goose adenovirus 1, aviadenovirus XZ03 (NCBI accession number PP319066.1 for DNA polymerase gene) as bar-headed Goose adenovirus 2, siadenoviruses clustered together as Group 1 of *Siadenovirus* in Figure 2 (e.g., XZ14, NCBI accession number PP319082.1 for DNA polymerase gene) as bar-headed Goose adenovirus 3, and barthadenovirus XZ13 (NCBI accession number PP319098.1 for DNA polymerase gene) as bar-headed Goose adenovirus 4.

In general, adenoviruses demonstrated host specificity [38]. The high sequence identity of majority of the siadenoviruses detected, mostly from *A. indicus* in this study indicated that these adenovirus strains were probably from the same or similar species. Although frog adenovirus with amphibians as hosts has been considered the common ancestor of the genus *Siadenovirus* [4], it has been controversial regarding the origin of siadenoviruses partly due to the lack of data from a broad range of hosts. Our work provided evidence for the existence of a wide range of unknown adenoviruses to trigger further investigation into the genome composition and structure of these potentially novel species, particularly those of more ancestral, such as *L. ridibundus* adenovirus YN22 for *Siadenovirus*, and ultimately potential pathogenesis for disease prevention and control.

The *Aviadenovirus* genus is usually considered a genus of bird adenoviruses. The main branch of the adenoviruses of *A. indicus* and *A. albifrons* in this genus suggested their shared origin with Duck aviadenovirus 2. More importantly, a cluster of sequences obtained from diverse bird species demonstrated moderate homology to FAdV A–E with an amino acid sequence identity of 60%–70%. In recent years, FAdV has been recognized as a threat to the poultry industry globally causing increased disease outbreaks, including China, although its pathogenesis is still unclear [39–42]. Carryover of FAdV-induced disease into wild birds has also been reported. Genomic characterization of these FAdV-like adenoviruses would help elucidate their evolutionary relationship and understand their potential virulence.

Despite the short DNA polymerase sequences used and the lack of epidemiological evidence for network analysis, it was still possible to understand the diversity of adenoviruses in birds. During the evolution of adenoviruses, host migration occurred due to the roosting, physical migration, and predation of birds and other animals, which contributed to the evolution of adenoviruses and the emergence of novel adenoviruses. Based on the phylogenetic tree and the NeighborNet analysis, our work expanded the host range of adenoviruses and revealed the relationship across lineages (Figure S2). The adenoviruses detected in one *P. carbo* and one *C. alpina* in this study were located in the reptile lineage of barthadenoviruses in the phylogenetic tree, which was rare in birds. Considering *P. carbo* is a carnivore animal, similar to the finding in Brazil [43], the adenoviruses found here might be a foodborne contaminant originating from the prey animal, likely a scaled reptile. Nonetheless, these could also suggest that host exchange might have occurred between different lineages during the evolution and that they possibly existed as an intermediate host, where the viruses had already adapted to.

Despite significant transmission constraints between different viral hosts, repeated mutations, co-circulation, and occasional gene flow have led to high local diversity of viral genomes [44]. Several recombination events were recognized in the hexon gene in our work, which is consistent with the notion that recombination is common in adenoviruses and plays a key role in the evolution of viruses and the emergence of novel viruses [45–50]. From the NeighborNet and recombination analysis, recombination events occurred across different locations and different migratory bird types, likely due to the shared habitats among species, particularly at mass migratory habitats, and frequent viral co-infection [51, 52]. Several mixed cases were identified in this study through next-generation sequencing, including both intra- and inter-genera, which would largely increase the probability of recombination and further lead to the emergence of novel species. More frequent observation of recombination in aviadenoviruses was partially biased by the acquisition of more hexon sequences from aviadenovirus positive samples. Nonetheless, it might also reflect the more frequent transmission of this genus between wild birds and poultry, with the evidence showing recombination product resulting from Duck adenovirus 2. To our knowledge, scattered small-scale poultry farms may be located in the vicinity of some habitats. Therefore, extensive surveillance of such recombination events in both wild birds and domestic animals is of great importance to understand the transmission risk from a poultry industry perspective.

Migration has long been proven to impact the transmission of avian pathogens. In the current study, adenoviruses were found in *A. indicus* from Qinghai and Xizang, *Anser fabalis* and *A. albifrons* from Heilongjiang and Inner Mongolia, and *L. ridibundus* from Yunnan Province. Based on the known migration routes [53], *A. indicus* overwinters in Xizang, and breeds in Qinghai and Mongolia [54]. Both *A. fabalis* and *A. albifrons* overwinter in Poyang Lake in Jiangxi and travel to Russia to breed via Heilongjiang. Aviadenoviruses were the most represented adenoviruses carried by these birds, of which Goose adenovirus 4 was the main adenovirus belonging to the species *Aviadenovirus anseris*. This type of adenovirus originated from goslings that died young, nonetheless it has not been reported to be pathogenic [55]. The migration routes of *A. indicus*, *A. fabalis*, and *A. albifrons* cover the eastern and western regions of China, in particular, the eastern region is on the East Asian migration line of Australia. The complex bird species and the overlapping migration routes could make it more conducive to the transmission of adenoviruses. *L. ridibundus* overwinters in Yunnan and travels to Russia via Hebei Province for breeding. Similarly, aviadenovirus was the main type of adenovirus carried by *L. ridibundus*, with sequence identity lower than 85% compared to the known sequences. Although the current study could not confirm whether it was a new adenovirus, its potential transmission should not be ignored, but promoting further investigation.

The main limitation of the current study was that the analysis was performed merely on DNA polymerase and hexon sequences. The attempt to amplify the flanking sequences was not successful due to the low identity to the known viral sequences. Further characterization of the novel species, particularly recombination analysis will rely on metagenomic sequencing and assembly, or viral culture followed by whole genome sequencing. Similarly, due to the high diversity of the adenoviruses, the hexon primers designed based on known adenoviruses failed to amplify for most of the samples, particularly siadenoviruses. The addition of one primer to the aviadenovirus assay improved the detection to some extent. Therefore, only a subset, mainly aviadenoviruses, were included in the phylogenetic comparison and recombination analysis. Additionally, analysis of seasonality across sites was limited because of the sample availability. After all, we were not able to systematically analyze the spatial and temporal carriage of adenoviruses by migratory birds.

## 5. Conclusions

Diverse and potentially novel adenoviruses, belonging to at least 10 species, were detected in 38 migratory bird species across nine habitats in China in the current study. Host exchange and recombination events were observed. The wide and abundant distribution of these adenoviruses highlighted the transmission between wild birds and poultry, potentially humans, and reinforced the one health approach for infectious disease control. Prospective and systematic pathogen surveillance would enable the tracking of temporal and geographical changes to explore the impact on pathogen carriage and spread by migratory birds, in particular the emergence of novel species or variants upon the change of animal behavior. Furthermore, metagenomic sequencing can provide a powerful tool to explore the genetic properties for a comprehensive view of such novel pathogen species.

## Figures and Tables

**Table 1:** Primer sequences used in this study.

Taxon	Primer sense	Primer (5'–3')	Target gene	Reference
Adenovirus	Forward 1	TNMGNGGNGGNMGNTGYTAYCC	DNA polymerase	[22]
	Reverse 1	GTDGCRAANSHNCCRTABARNGMRTT	DNA polymerase	[22]
	Forward 2	GTNTWYGAYATHTGYGGHATGTAYGC	DNA polymerase	[22]
	Reverse 2	CCANCCBCDRTTRTGNARNGTRA	DNA polymerase	[22]

*Aviadenovirus*	Forward 1	TATTTTCACATYGCSGGCCC	Hexon	This study
	Forward 2	YAARTTYAGRCAGACGGT	Hexon	[23]
	Reverse	TAGTGATGHCKBGACATCAT	Hexon	[23]

*Siadenovirus*	Forward	CAYATAGCTGGACMAGAYGC	Hexon	This study
	Reverse	TAATCATCWACAGCYTGRTTCCA	Hexon	This study

Bird	Forward	CACGAATAAACAACATAAGCTTCTG	COI	[24]
	Reverse 1	CAGGGTGTCCGAAGAATC	COI	[25]
	Reverse 2	CTGGGTGGCCRAARAATC	COI	This study

**Table 2:** Detection of recombination events based on hexon gene.

Recombination	Major parent	Minor parent	Detection method^a^						
			R	G	B	M	C	S	T
*Anser indicus* adenovirus XZ04	*Anser indicus* adenovirus XZ06	*Anser albifrons* adenovirus HLJ13	+	+	−	−	−	−	+
*Anser albifrons* adenovirus HLJ13	*Anser indicus* adenovirus QH01	*Anser indicus* adenovirus XZ04	+	+	+	+	+	−	−
*Larus ridibundus* adenovirus YN21	*Anser albifrons* adenovirus HLJ21	CsPADV2	+	−	+	+	+	+	−
*Cygnus columbianus* adenovirus FJ06	*Anser indicus* adenovirus HeB08	*Anser albifrons* adenovirus HLJ21	+	−	−	−	−	−	+
GooseADV4	*Anser indicus* adenovirus QH01	*Anser indicus* adenovirus XZ02	+	+	+	+	+	−	−
PsiADV4	PigeonADV1	RaptorADVA	−	−	−	+	+	+	−
BatADV3	OvineADV8	DuckADV2	−	−	−	+	+	+	−
*Cygnus columbianus adenovirus* FJ06	DuckADV2	*Anser indicus* adenovirus XZ02	−	+	+	−	−	+	−
*Anser albifrons* adenovirus HLJ11	*Anser indicus* adenovirus XZ02	*Anser indicus* adenovirus XZ12	−	−	−	−	−	+	+
*Anser indicus* adenovirus XZ03	*Anser albifrons* adenovirus HLJ12	*Anser indicus* adenovirus XZ04	−	+	−	−	−	+	−
PigeonADV2	TurkeyADV1	FrogADV1	−	−	−	−	−	+	+

*Note*: Recombination analysis was performed using RDP4 software. Recombination event was counted when supported by at least two methods.

^a^R, RDP; G, GENECONV; B, Bootscan; M, MaxChi; C, Chimaera; S, Siscan; T, 3Seq methods.

**Table 3:** Cluster analysis of potentially novel adenoviruses.

Genus	Species	Number of sequences per location									Number per species
		Xizang	Qinghai	Hebei	Heilongjiang	Yunnan	Fujian	Hubei	Ningxia	Inner Mongolia	
*Aviadenovirus*	Species 1					9					9
	Species 2					4					4
	Species 3			1					1		2
	Species 4					2					2
	Species 5		1						1		2
	Species 6			1			1				2
	Species 7								2		2
	Other species^a^ (*n* = 15)	2		3		1	3	3	3		1

*Siadenovirus*	Species 1	17	1		8			2		1	29
	Species 2			2							2
	Other species (*n* = 10)	2		1		4	2			1	1

*Barthadenovirus*	Species 1			2			1		2		5
	Other species (*n* = 4)	1					1		2		1
Total		22	2	10	8	20	8	5	11	2	

*Note*: A species was identified for sequences with >85% identity.

^a^Other species refers to those that had only one sequence.

## Data Availability

The sequences generated and presented in this study have been deposited in NCBI GenBank under accession numbers PP319046–PP319176 and PP331810–PP331835. The data that support the findings of this study are available from the corresponding author upon reasonable request.

## Supporting Information

Supporting Information Table S1. The distribution of adenoviruses in migratory birds in this study. Table S2. The information of sequences generated in the current study that were used for SplitsTree analysis. Table S3. The information of sequences from the NCBI GenBank used for SplitsTree analysis. Figure S1. Visualization of recombination analysis on adenovirus hexon gene sequences. Figure S2. NeighborNet analysis using nucleotide sequences of DNA polymerase gene. Adenoviruses in the same avian order or vertebrate class are marked by the same color. The sequences of adenoviruses detected in this study are marked by dots (•), and different colored circles represent different lineages. Sequence information of adenoviruses is listed in Supporting Table S2 and S3.
