# Supplementary material for: Molecular Characterization Reveals Recombination and Host Exchange of Adenoviruses in Migratory Birds in China
Source: Transbound Emerg Dis. 2025 Sep 27;2025:3030377. doi: 10.1155/tbed/3030377 (PMC12496158; doi:10.1155/tbed/3030377)

Table S1. The distribution of adenoviruses in migratory birds in this study

| **Location** | **Bird Order** | **Bird Family** | **Adenovirus genus** | | | **Negative** | **Total^1^** |
| --- | --- | --- | --- | --- | --- | --- | --- |
|  |  |  | ***Aviadenovirus*** | ***Siadenovirus*** | ***Barthadenovirus*** |  |  |
| Fujian | Anseriformes | Anatidae | 2 | 0 | 1 | 14 | 17 |
|  | Charadriformes | Charadriidae | 0 | 0 | 0 | 1 | 1 |
|  |  | Laridae | 1 | 0 | 0 | 0 | 1 |
|  |  | Scolopacidae | 1 | 1 | 1 | 18 | 21 |
|  |  | Unidentified | 1 | 1 | 0 | 5 | 7 |
| Hebei | Anseriformes | Anatidae | 2 | 0 | 0 | 9 | 11 |
|  | Charadriformes | Recurvirostridae | 1 | 2 | 0 | 2 | 5 |
|  |  | Charadriidae | 0 | 0 | 0 | 2 | 2 |
|  |  | Laridae | 3 | 0 | 0 | 19 | 22 |
|  |  | Glareolidae | 0 | 0 | 0 | 1 | 1 |
|  |  | Scolopacidae | 0 | 0 | 0 | 5 | 5 |
|  | Suliformes | Phalacrocoracidae | 0 | 1 | 0 | 3 | 4 |
|  | Strigiformes | Strigidae | 1 | 0 | 0 | 1 | 2 |
|  |  | Unidentified | 2 | 0 | 2 | 36 | 40 |
| Heilongjiang | Anseriformes | Anatidae | 18 | 8 | 0 | 60 | 86 |
| Hubei | Anseriformes | Anatidae | 4 | 2 | 0 | 36 | 41* |
|  | Pelecaniformes | Ardeidae | 1 | 0 | 0 | 0 | 1 |
|  |  | Unidentified | 1 | 0 | 0 | 3 | 4 |
| Inner Mongolia | Anseriformes | Anatidae | 2 | 2 | 0 | 41 | 45 |
|  | Gruiformes | Gruidae | 0 | 0 | 0 | 1 | 1 |
| Ningxia | Anseriformes | Anatidae | 1 | 0 | 0 | 11 | 12 |
|  | Charadriformes | Recurvirostridae | 1 | 0 | 0 | 6 | 7 |
|  |  | Charadriidae | 4 | 0 | 1 | 8 | 11* |
|  |  | Laridae | 0 | 0 | 0 | 2 | 2 |
|  |  | Scolopacidae | 1 | 0 | 3 | 2 | 5* |
|  | Pelecaniformes | Ardeidae | 0 | 0 | 0 | 1 | 1 |
|  | Gruiformes | Rallidae | 0 | 0 | 0 | 1 | 1 |
|  |  | Unidentified | 0 | 0 | 0 | 7 | 7 |
| Qinghai | Anseriformes | Anatidae | 2 | 1 | 0 | 47 | 50 |
| Tibet | Anseriformes | Anatidae | 14 | 19 | 0 | 189 | 222 |
|  | Suliformes | Phalacrocoracidae | 0 | 0 | 1 | 0 | 1 |
| Yunnan | Charadriformes | Laridae | 16 | 6 | 0 | 32 | 54 |
| Total^2^ |  |  | **79** | **43** | **9** | **563** | **690*** |

* Presence of mixed infections/carriages of adenoviruses

^1^ The number of samples

^2^ The number of adenovirus sequences

Table S2. The information of sequences determined in the current study that were used for Splite tree analysis.

| **ID** | **Location** | **Bird species** | **Bird family** | **Adenovirus genus** | **Name in Figure 2** | **Name in Figure S2** |
| --- | --- | --- | --- | --- | --- | --- |
| 1 | Hebei | *Anser indicus* | Anatidae | *Aviadenovirus* | HeB08 | Anser29 |
| 2 | Hebei | *Recurvirostra avosetta* | Recurvirostridae | *Aviadenovirus* | HeB06 | Recurvirostra01 |
| 3 | Hebei | *Asio flammeus* | Strigidae | *Aviadenovirus* | - | Aiso01 |
| 4 | Hebei | Unidentified | Unidentified | *Aviadenovirus* | HeB09 | Unidentified03 |
| 5 | Hebei | Unidentified | Unidentified | *Aviadenovirus* | HeB10 | Unidentified04 |
| 6 | Hebei | *Phalacrocorax carbo* | Phalacrocoracidae | *Siadenovirus* | HeB13 | Phalacrocorax01 |
| 7 | Heilongjiang | *Anas platyrhynchos* | Anatidae | *Aviadenovirus* | HLJ14 | Anas03 |
| 8 | Heilongjiang | *Anser albifrons* | Anatidae | *Aviadenovirus* | HLJ10 | Anser01 |
| 9 | Heilongjiang | *Anser albifrons* | Anatidae | *Aviadenovirus* | HLJ11 | Anser02 |
| 10 | Heilongjiang | *Anser albifrons* | Anatidae | *Aviadenovirus* | HLJ12 | Anser03 |
| 11 | Heilongjiang | *Anas platyrhynchos* | Anatidae | *Aviadenovirus* | HLJ15 | Anas04 |
| 12 | Heilongjiang | *Anser albifrons* | Anatidae | *Aviadenovirus* | HLJ13 | Anser04 |
| 13 | Heilongjiang | *Anser albifrons* | Anatidae | *Siadenovirus* | HLJ21 | Anser05 |
| 14 | Heilongjiang | *Anser albifrons* | Anatidae | *Siadenovirus* | HLJ23 | Anser06 |
| 15 | Heilongjiang | *Anser albifrons* | Anatidae | *Siadenovirus* | HLJ22 | Anser07 |
| 16 | Heilongjiang | *Anser albifrons* | Anatidae | *Siadenovirus* | HLJ20 | Anser08 |
| 17 | Heilongjiang | *Anser albifrons* | Anatidae | *Aviadenovirus* | HLJ01 | Anser09 |
| 18 | Heilongjiang | *Anser albifrons* | Anatidae | *Aviadenovirus* | HLJ02 | Anser10 |
| 19 | Heilongjiang | *Anser albifrons* | Anatidae | *Siadenovirus* | HLJ16 | Anser11 |
| 20 | Heilongjiang | *Anser albifrons* | Anatidae | *Siadenovirus* | HLJ17 | Anser12 |
| 21 | Heilongjiang | *Anser albifrons* | Anatidae | *Aviadenovirus* | - | Anser13 |
| 22 | Heilongjiang | *Anser albifrons* | Anatidae | *Aviadenovirus* | - | Anser14 |
| 23 | Heilongjiang | *Anser albifrons* | Anatidae | *Aviadenovirus* | HLJ03 | Anser15 |
| 24 | Heilongjiang | *Anser albifrons* | Anatidae | *Siadenovirus* | HLJ18 | Anser16 |
| 25 | Heilongjiang | *Anser albifrons* | Anatidae | *Aviadenovirus* | HLJ04 | Anser17 |
| 26 | Heilongjiang | *Anser albifrons* | Anatidae | *Aviadenovirus* | - | - |
| 27 | Heilongjiang | *Anser albifrons* | Anatidae | *Aviadenovirus* | HLJ05 | Anser18 |
| 28 | Heilongjiang | *Anser albifrons* | Anatidae | *Aviadenovirus* | HLJ06 | Anser19 |
| 29 | Heilongjiang | *Anser albifrons* | Anatidae | *Siadenovirus* | HLJ19 | Anser20 |
| 30 | Heilongjiang | *Anser albifrons* | Anatidae | *Aviadenovirus* | HLJ07 | Anser21 |
| 31 | Heilongjiang | *Anser albifrons* | Anatidae | *Aviadenovirus* | HLJ08 | Anser22 |
| 32 | Heilongjiang | *Anser albifrons* | Anatidae | *Aviadenovirus* | HLJ09 | Anser23 |
| 33 | Qinghai | *Aythya nyroca* | Anatidae | *Aviadenovirus* | QH01 | Aythya01 |
| 34 | Qinghai | *Anser indicus* | Anatidae | *Aviadenovirus* | QH02 | Anser33 |
| 35 | Qinghai | *Anser indicus* | Anatidae | *Siadenovirus* | QH03 | Anser34 |
| 36 | Tibet | *Anser indicus* | Anatidae | *Aviadenovirus* | XZ01 | Anser35 |
| 37 | Tibet | *Anser indicus* | Anatidae | *Aviadenovirus* | XZ06 | Anser36 |
| 38 | Tibet | *Anser indicus* | Anatidae | *Aviadenovirus* | XZ10 | Anser37 |
| 39 | Tibet | *Anser indicus* | Anatidae | *Aviadenovirus* | XZ11 | Anser38 |
| 40 | Tibet | *Anser indicus* | Anatidae | *Aviadenovirus* | XZ04 | Anser39 |
| 41 | Tibet | *Anser indicus* | Anatidae | *Aviadenovirus* | XZ02 | Anser40 |
| 42 | Tibet | *Anser indicus* | Anatidae | *Aviadenovirus* | - | Anser41 |
| 43 | Tibet | *Anser anser* | Anatidae | *Aviadenovirus* | XZ12 | Anser24 |
| 44 | Hebei | *Anser indicus* | Anatidae | *Aviadenovirus* | HeB07 | Anser30 |
| 45 | Tibet | *Anser indicus* | Anatidae | *Aviadenovirus* | XZ07 | Anser42 |
| 46 | Tibet | *Anser indicus* | Anatidae | *Aviadenovirus* | XZ03 | Anser43 |
| 47 | Tibet | *Anser indicus* | Anatidae | *Aviadenovirus* | - | Anser44 |
| 48 | Tibet | *Anser indicus* | Anatidae | *Aviadenovirus* | XZ05 | Anser45 |
| 49 | Tibet | *Anser indicus* | Anatidae | *Aviadenovirus* | XZ08 | Anser46 |
| 50 | Tibet | *Anser indicus* | Anatidae | *Aviadenovirus* | XZ09 | Anser47 |
| 51 | Tibet | *Phalacrocorax carbo* | Phalacrocoracidae | *Barthadenovirus* | XZ13 | Phalacrocorax02 |
| 52 | Tibet | *Anser indicus* | Anatidae | *Siadenovirus* | XZ28 | Anser48 |
| 53 | Tibet | *Anser indicus* | Anatidae | *Siadenovirus* | XZ19 | Anser49 |
| 54 | Tibet | *Anser indicus* | Anatidae | *Siadenovirus* | - | Anser50 |
| 55 | Tibet | *Anser indicus* | Anatidae | *Siadenovirus* | XZ25 | Anser51 |
| 56 | Tibet | *Anser indicus* | Anatidae | *Siadenovirus* | XZ14 | Anser52 |
| 57 | Tibet | *Anser indicus* | Anatidae | *Siadenovirus* | XZ15 | Anser53 |
| 58 | Tibet | *Anser indicus* | Anatidae | *Siadenovirus* | XZ27 | Anser54 |
| 59 | Tibet | *Anser indicus* | Anatidae | *Siadenovirus* | XZ24 | Anser55 |
| 60 | Tibet | *Anser indicus* | Anatidae | *Siadenovirus* | XZ16 | Anser56 |
| 61 | Tibet | *Anser indicus* | Anatidae | *Siadenovirus* | XZ17 | Anser57 |
| 62 | Tibet | *Anser indicus* | Anatidae | *Siadenovirus* | XZ22 | Anser58 |
| 63 | Tibet | *Anser indicus* | Anatidae | *Siadenovirus* | XZ18 | Anser59 |
| 64 | Tibet | *Anser indicus* | Anatidae | *Siadenovirus* | XZ20 | Anser60 |
| 65 | Tibet | *Anser indicus* | Anatidae | *Siadenovirus* | XZ21 | Anser61 |
| 66 | Tibet | *Anser indicus* | Anatidae | *Siadenovirus* | - | Anser62 |
| 67 | Tibet | *Anser indicus* | Anatidae | *Siadenovirus* | - | Anser63 |
| 68 | Tibet | *Anser indicus* | Anatidae | *Siadenovirus* | - | Anser64 |
| 69 | Tibet | *Anser indicus* | Anatidae | *Siadenovirus* | XZ23 | Anser65 |
| 70 | Tibet | *Anser indicus* | Anatidae | *Siadenovirus* | XZ26 | Anser66 |
| 71 | Hebei | *Himantopus himantopus* | Recurvirostridae | *Siadenovirus* | HeB11 | Himantopus02 |
| 72 | Hebei | *Ichthyaetus relictus* | Laridae | *Aviadenovirus* | HeB03 | Ichthyaetus01 |
| 73 | Hebei | *Ichthyaetus relictus* | Laridae | *Aviadenovirus* | HeB04 | Ichthyaetus02 |
| 74 | Hebei | *Himantopus himantopus* | Recurvirostridae | *Siadenovirus* | HeB12 | Himantopus01 |
| 75 | Hebei | Unidentified | Unidentified | *Barthadenovirus* | HeB01 | Unidentified05 |
| 76 | Hebei | Unidentified | Unidentified | *Barthadenovirus* | HeB02 | Unidentified06 |
| 77 | Hebei | *Larus crassirostris* | Laridae | *Aviadenovirus* | HeB05 | Larus01 |
| 78 | Yunnan | *Larus ridibundus* | Laridae | *Aviadenovirus* | YN01 | Larus03 |
| 79 | Yunnan | *Larus ridibundus* | Laridae | *Aviadenovirus* | YN02 | Larus04 |
| 80 | Yunnan | *Larus ridibundus* | Laridae | *Siadenovirus* | YN17 | Larus05 |
| 81 | Yunnan | *Larus ridibundus* | Laridae | *Aviadenovirus* | YN03 | Larus06 |
| 82 | Yunnan | *Larus ridibundus* | Laridae | *Aviadenovirus* | YN04 | Larus07 |
| 83 | Yunnan | *Larus ridibundus* | Laridae | *Aviadenovirus* | YN05 | Larus08 |
| 84 | Yunnan | *Larus ridibundus* | Laridae | *Siadenovirus* | YN18 | Larus09 |
| 85 | Yunnan | *Larus ridibundus* | Laridae | *Siadenovirus* | YN19 | Larus10 |
| 86 | Yunnan | *Larus ridibundus* | Laridae | *Aviadenovirus* | YN06 | Larus11 |
| 87 | Yunnan | *Larus ridibundus* | Laridae | *Siadenovirus* | YN20 | Larus12 |
| 88 | Yunnan | *Larus ridibundus* | Laridae | *Siadenovirus* | YN21 | Larus13 |
| 89 | Yunnan | *Larus ridibundus* | Laridae | *Aviadenovirus* | YN07 | Larus14 |
| 90 | Yunnan | *Larus ridibundus* | Laridae | *Aviadenovirus* | YN08 | Larus15 |
| 91 | Yunnan | *Larus ridibundus* | Laridae | *Aviadenovirus* | YN09 | Larus16 |
| 92 | Yunnan | *Larus ridibundus* | Laridae | *Aviadenovirus* | YN10 | Larus17 |
| 93 | Yunnan | *Larus ridibundus* | Laridae | *Aviadenovirus* | YN11 | Larus18 |
| 94 | Yunnan | *Larus ridibundus* | Laridae | *Aviadenovirus* | YN12 | Larus19 |
| 95 | Yunnan | *Larus ridibundus* | Laridae | *Aviadenovirus* | YN13 | Larus20 |
| 96 | Yunnan | *Larus ridibundus* | Laridae | *Aviadenovirus* | YN14 | Larus21 |
| 97 | Yunnan | *Larus ridibundus* | Laridae | *Aviadenovirus* | YN15 | Larus22 |
| 98 | Yunnan | *Larus ridibundus* | Laridae | *Siadenovirus* | YN22 | Larus23 |
| 99 | Yunnan | *Larus ridibundus* | Laridae | *Aviadenovirus* | YN16 | Larus24 |
| 100 | Fujian | *Numenius arquata* | Scolopacidae | *Aviadenovirus* | FJ02 | Numenius01 |
| 101 | Fujian | *Calidris alpina* | Scolopacidae | *Barthadenovirus* | FJ07 | Calidris01 |
| 102 | Fujian | *Larus fuscus* | Laridae | *Aviadenovirus* | FJ03 | Larus02 |
| 103 | Fujian | Unidentified | Unidentified | *Aviadenovirus* | FJ04 | Unidentified01 |
| 104 | Fujian | *Anas falcata* | Anatidae | *Aviadenovirus* | FJ05 | Anas01 |
| 105 | Fujian | Unidentified | Unidentified | *Siadenovirus* | FJ08 | Unidentified02 |
| 106 | Fujian | *Cygnus columbianus* | Anatidae | *Aviadenovirus* | FJ06 | Cygnus02 |
| 107 | Fujian | *Numenius arquata* | Scolopacidae | *Siadenovirus* | FJ09 | Numenius02 |
| 108 | Fujian | *Anas falcata* | Anatidae | *Barthadenovirus* | FJ01 | Anas02 |
| 109 | Ningxia | *Recurvirostra avosetta* | Recurvirostridae | *Aviadenovirus* | NX05 | Recurvirostra02 |
| 110 | Ningxia | *Philomachus pugnax* | Scolopacidae | *Barthadenovirus* | NX03 | Philomachus01 |
| 111 | Ningxia | *Philomachus pugnax* | Scolopacidae | *Aviadenovirus* | NX09 | Philomachus02 |
| 112 | Ningxia | *Tringa erythropus* | Scolopacidae | *Barthadenovirus* | NX01 | Tringa01 |
| 113 | Ningxia | *Tringa erythropus* | Scolopacidae | *Barthadenovirus* | NX02 | Tringa02 |
| 114 | Ningxia | *Charadrius placidus* | Charadriidae | *Aviadenovirus* | NX06 | Charadrius01 |
| 115 | Ningxia | *Charadrius placidus* | Charadriidae | *Aviadenovirus* | NX10 | Charadrius02 |
| 116 | Ningxia | *Charadrius placidus* | Charadriidae | *Aviadenovirus* | NX11 | Charadrius03 |
| 117 | Ningxia | *Charadrius placidus* | Charadriidae | *Barthadenovirus* | NX04 | Charadrius04 |
| 118 | Ningxia | *Netta rufina* | Anatidae | *Aviadenovirus* | NX07 | Netta01 |
| 119 | Ningxia | *Pluvialis fulva* | Charadriidae | *Aviadenovirus* | NX08 | Pluvialis01 |
| 120 | Hubei | *Anser fabalis* | Anatidae | *Siadenovirus* | - | - |
| 121 | Hubei | *Anser cygnoides* | Anatidae | *Aviadenovirus* | - | - |
| 122 | Hubei | *Anser fabalis* | Anatidae | *Aviadenovirus* | HuB01 | Anser26 |
| 123 | Hubei | *Anser indicus* | Anatidae | *Aviadenovirus* | HuB05 | Anser31 |
| 124 | Hubei | *Anser indicus* | Anatidae | *Siadenovirus* | HuB06 | Anser32 |
| 125 | Hubei | *Ardea alba* | Ardeidae | *Aviadenovirus* | HuB02 | Ardea01 |
| 126 | Hubei | Unidentified | Unidentified | *Aviadenovirus* | HuB03 | Unidentified07 |
| 127 | Hubei | *Cygnus bewickii* | Anatidae | *Aviadenovirus* | HuB04 | Cygnus01 |
| 128 | Inner Mongolia | *Anser fabalis* | Anatidae | *Siadenovirus* | NMG02 | Anser27 |
| 129 | Inner Mongolia | *Anser fabalis* | Anatidae | *Siadenovirus* | NMG03 | Anser28 |
| 130 | Inner Mongolia | *Anser cygnoides* | Anatidae | *Aviadenovirus* | NMG01 | Anser25 |
| 131 | Inner Mongolia | *Anser fabalis* | Anatidae | *Aviadenovirus* | - | - |

Table S3. The information of sequences from the NCBI GenBank used for Splits tree analysis.

| Abbreviation used in the splits tree | Adenovirus name | GenBank accession number |
| --- | --- | --- |
| canine 1 | Canine adenovirus type 1 | U55001.1 |
| frog | Frog adenovirus 1 | NC_002501.1 |
| ovine 7 | Ovine adenovirus 7 | U40839.3 |
| bovine 4 | Bovine adenovirus 4 | AF036092.3 |
| simian 3 | Simian adenovirus 3 | AY598782.1 |
| ovine A | Ovine adenovirus A | AC_000001.1 |
| human A | Human mastadenovirus A | AC_000005.1 |
| murine 1 | Murine adenovirus 1 | AC_000012.1 |
| fowl A | Fowl aviadenovirus A | AC_000014.1 |
| turkey A | Turkey siadenovirus A | AC_000016.1 |
| murine 3 | Murine adenovirus 3 | EU835513.1 |
| great tit | Great tit siadenovirus A | FJ849795.1 |
| fowl C | Fowl aviadenovirus C | GU188428.1 |
| murine 2 | Murine adenovirus 2 | HM049560.1 |
| raptor 1 | Raptor adenovirus 1 | NC_015455.1 |
| skua 1 | South polar skua adenovirus-1 | HM585353.1 |
| turkey 1 | Turkey adenovirus 1 | GU936707.2 |
| goose 4 | Goose adenovirus 4 | JF510462.1 |
| fowl 5 | Fowl adenovirus 5 | KC493646.1 |
| sea lion | California sea lion adenovirus 1 | KJ563221.1 |
| lizard 2 | Lizard adenovirus 2 | KJ156523.1 |
| psittacine 3 | Psittacine adenovirus 3 | KJ675568.1 |
| skunk | Skunk adenovirus PB1 | KP238322.1 |
| penguin | Penguin siadenovirus A | KP144329.1 |
| bat | Bat adenovirus TMJ | GU226970.2 |
| fowl 6 | Fowl adenovirus 6 | KT862808.1 |
| pigeon 2 | Pigeon adenovirus 2 | KX121164.1 |
| psittacine B | Psittacine aviadenovirus B | KX577802.1 |
| deer A | Odocoileus adenovirus A | NC_035619.1 |
| psittacine 1 | Psittacine adenovirus 1 | MH580295.1 |
| psittacine 3 type 1 | Psittacine 3 type 1 | MN025529.1 |
| amniota | Amniota adenovirus | MN025530.1 |
| white-eyed parrot | Southern Psittacara leucophthalmus aviadenovirus 1 | MN153802.1 |
| pacific parrotlet | Siadenovirus IDL-3602 | MK695679.1 |
| l. corella | Little corella adenovirus | MK227353.1 |
| eublepharid | Eublepharid adenovirus 1 | AY576677.1 |
| chameleon | Chameleon adenovirus 1 | AY576679.1 |
| helodermatid | Helodermatid adenovirus 1 | AY576680.1 |
| gekkonid | Gekkonid adenovirus 1 | AY576681.1 |
| scincid 1 | Scincid adenovirus 1 | AY576682.1 |
| Meyer’s parrot | Meyer’s parrot adenovirus 1 | AY644731.1 |


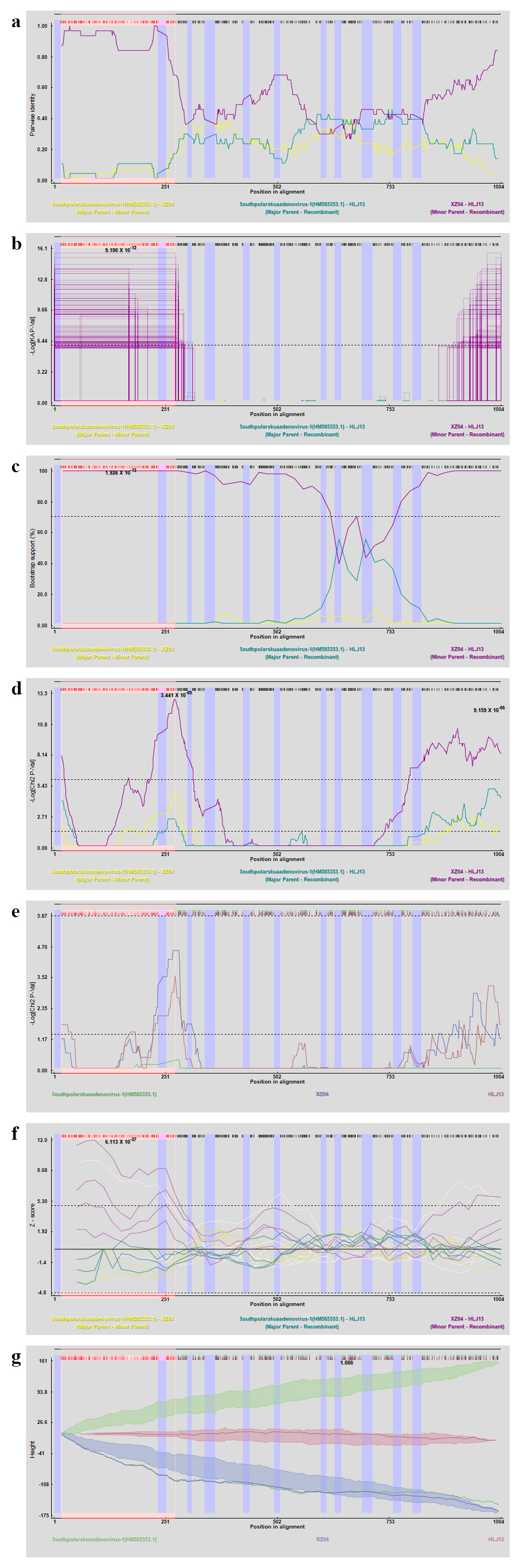
Figure S1. Visualization of recombination analysis on adenovirus hexon gene sequences, with schematic sequence displays for all the interrogated sequences (left) and plot displays for *Anser albifrons* adenovirus HLJ13 as an example (right). The plot display a through g represents 7 methods, i.e. RDP, GENECONV, Bootscan, MaxChi, Chimaera, Siscan, and 3Seq.

Figure S2 NeighborNet analysis using nucleotide sequences of DNA polymerase gene. Adenoviruses in the same avian order or vertebrate class are marked by the same color. The sequences of adenoviruses detected in this study are marked by dots (•), and different colored circles represent different lineages. Sequence information of adenoviruses is listed in Supplementary Table S2 & S3.


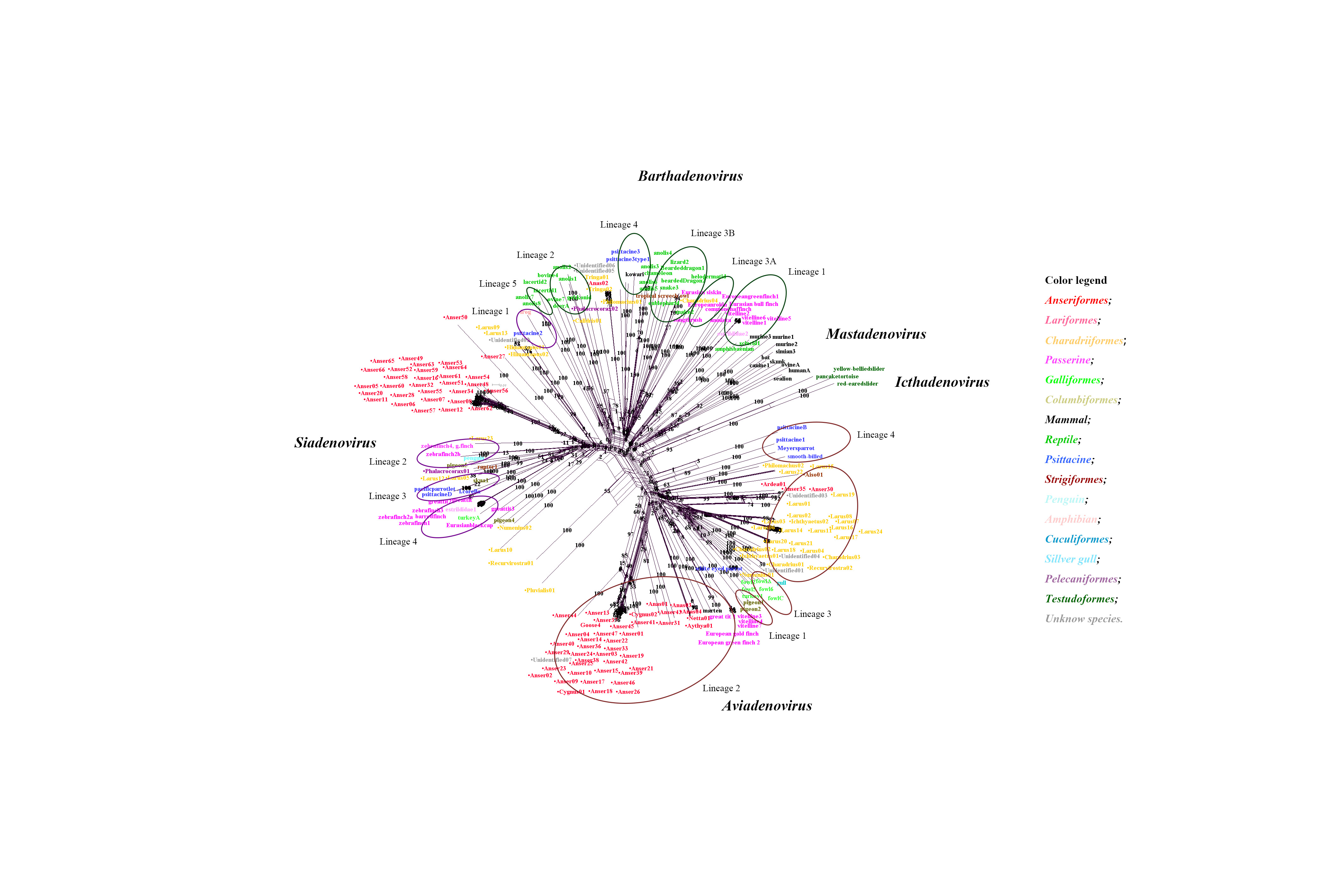

Supplement: Supporting Information — Table S1. The distribution of adenoviruses in migratory birds in this study. Table S2. The information of sequences generated in the current study that were used for SplitsTree analysis. Table S3. The information of sequences from the NCBI GenBank used for SplitsTree analysis. Figure S1. Visualization of recombination analysis on adenovirus hexon gene sequences. Figure S2. NeighborNet analysis using nucleotide sequences of DNA polymerase gene. Adenoviruses in the same avian order or vertebrate class are marked by the same color. The sequences of adenoviruses detected in this study are marked by dots (•), and different colored circles represent different lineages. Sequence information of adenoviruses is listed in Supporting Table S2 and S3. [file 3030377.f1.docx]
